# First-line carboplatin-based chemotherapy may be beneficial for HER2-low advanced breast cancer: A retrospective analysis

**DOI:** 10.1097/MD.0000000000041082

**Published:** 2024-12-27

**Authors:** Jingxin Li, Yijing Tang, Qianying Chen, Sen Lei, Yongkui Lu, Aihua Tan, Weimin Xie

**Affiliations:** a Department of Breast, Bone & Soft Tissue Oncology, Guangxi Medical University Cancer Hospital, Nanning, China; b Graduate School of Guangxi Medical University, Nanning, China.

**Keywords:** breast cancer, carboplatin, chemotherapy, HER2-low, inflammatory index

## Abstract

For patients with human epidermal growth factor receptor 2 (HER2)-low advanced breast cancer who had failed to meet with anthracycline or taxane, the application of HER2-targeted antibody-drug conjugates as second-line therapy could improve patients' outcomes, but it is unclear whether carboplatin-based first-line therapy will benefit these patients. This retrospective study was designed to explore whether carboplatin based first-line treatment could improve outcomes in HER2-low advanced breast cancer, and to analyze potential factors affecting efficacy and prognosis. 103 patients with HER2-negative metastatic breast cancer were treated with carboplatin based first-line therapy. The differences in progression-free survival (PFS), objective response rate (ORR), and adverse events were analyzed in different HER2 expression subgroups. The risk ratio (HR) and 95% confidence interval (CI) for PFS were estimated using Cox proportional risk models. The ORR for the whole group was 42.72% and the median PFS (mPFS) was 7.93 months (m). The ORR of HER2-low patients was significantly higher than HER2-zero patients (56.4% vs 27.1%, *P* = .003), and HER2-zero was an independent risk factor of ORR (OR 3.478, 95%CI 1.516–7.977, *P* = .003), especially in the HR-negative subgroup. The mPFS was significantly longer in patients with low neutrophil-to-lymphocyte ratio (NLR) scores than those with high NLR scores (*P* < .001). Multivariate analysis showed that young breast cancer (age < 40) (*P* = .006) and high NLR values (*P* = .001) were prognostic risk factors affecting mPFS. The main grade 3 to 4 adverse reactions were neutropenia (15.53%), anemia (15.53%), and leukopenia (11.65%). The first-line carboplatin-based chemotherapy is quite active and tolerable in patients with HER2-low advanced breast cancer, that higher response rates can be achieved. In cases where CDK4/6 inhibitors are inappropriate for use due to resistance to endocrine therapy or the urgent need for short-term clinical response, chemotherapy remains important. When it is necessary to consider the accessibility of antibody-drug conjugates and the economics of patients, carboplatin-based chemotherapy may be provided to HER2-low patients as a more convenient, cost-effective and efficient option on the front line. Forecasting the efficacy and prognosis via inflammatory index such as NLR before the commencement of the treatment could enhance the precision and efficiency of carboplatin-based regimens.

## 1. Introduction

Breast cancer is one of the most common malignant tumors that seriously threaten women’s health. Globally, approximately 2.3 million new female breast cancer cases were estimated to have occurred in 2020.^[1]^ As chemotherapy is an important regimen for first-line therapy, the preferred agents recommended by the main guidelines in the field of breast cancer^[2,3]^ are anthracyclines and taxanes (single or sequential use), and other optional agents include capecitabine, vinorelbine, gemcitabine and platinum. However, the guidelines do not specify which class of agents should be preferred in the selection of capecitabine, vinorelbine, gemcitabine, and platinum. There are three main situations in which platinum-based agents are used in first-line chemotherapy. Firstly, triple-negative breast cancer (TNBC) with BRCA1/2 mutation. Secondly, platinum, taxanes and trastuzumab (TCbH) may be one of the preferred regimens for HER-2-positive advanced breast cancer in cases where pertuzumab are not available. Finally, platinum can be used for HER2-negative advanced breast cancer that has failed treatment with anthracyclines and taxanes, including TNBC and hormone receptor (HR)-positive advanced breast cancer with visceral metastases and obvious clinical symptoms or resistance to endocrine therapy.^[2,3]^

DESTINY-Breast04 ClinicalTrials found that patients with HER2-low breast cancer have benefited from the antibody-drug conjugates (ADCs) namely trastuzumab deruxtecan, rather than the physician’s choice of chemotherapy,^[4]^ which opened up new therapeutic ideas for HER2-low metastatic breast cancer. However, there are still no relevant studies on whether HER2-low patients can benefit from platinum-based chemotherapy regimens when ADCs are inaccessible, or cyclin-dependent kinase 4 and 6 (CDK4/6) inhibitors are inappropriate for use.

This study aimed to evaluate the efficacy, survival and safety of carboplatin based first-line chemotherapy in advanced breast cancer of HER2-low and HER2-zero, to explore whether carboplatin based first-line therapy could improve outcomes in HER2-low patients, and to analyze the potential factors affecting efficacy and prognosis.

## 2. Methods

### 2.1. Study design

This study enrolled a total of 103 HER2-negative metastatic breast cancer patients in the Guangxi Medical University Cancer Hospital from December 2013 to July 2020. All patients with a median age of 50 years (range 23–75 years) received carboplatin-based combination regimens as first-line rescue chemotherapy. The study was approved by the Ethics Committee of the Guangxi Medical University Cancer Hospital and was conducted in accordance with the 1964 Helsinki Declaration and its later amendments or comparable ethical standards.

### 2.2. Patient inclusion criteria

Diagnosed as invasive breast cancer by pathological biopsy with a complete molecular typing data including estrogen receptor (ER), progesterone receptor (PR), HER2 and Ki-67. HER2-zero is defined as IHC 0, HER2-low is defined as IHC 1+, or IHC 2+/ISH-. Diagnosed as metastatic breast cancer by clinical and imaging examinations. Age ≥ 18 years old. ECOG score ⩽ 2 and estimated survival ≥ 3 months. Received carboplatin-based combination regimens as first-line rescue therapy at least 2 cycles. Conducted efficacy evaluation and hematological toxicity evaluation. Complete blood count data available within 7 days prior to initiation of chemotherapy. At least one measurable lesion according to RECIST1.1 evaluation criteria. Normal results of electrocardiogram, routine blood test and liver/kidney function. No obvious contraindications for chemotherapy. Signed informed consent for chemotherapy.

### 2.3. Patient exclusion criteria

Locally advanced and non-metastatic breast cancer. Imperfect clinical, imaging or pathological data. Platinum was not included in first-line chemotherapy regimens. Failure to complete the prescribed chemotherapy cycle or failure to evaluate the efficacy as required. Have uncontrollable heart, liver, kidney, and other organic lesions, or combined with serious infections, cardiovascular and cerebrovascular diseases, respiratory diseases, etc.

### 2.4. Treatment methods

The carboplatin-based combination regimens in this study included carboplatin combined with docetaxel in 79 patients, paclitaxel liposomes in 22 patients and albumin paclitaxel in 2 patients. 20 of 103 patients were treated with the antiangiogenic rh-endostatin or bevacizumab. The dosage and usage of each drug were as follows, carboplatin AUC = 5 or 400 mg/m^2^, intravenous drip, d1; docetaxel 75 mg/m^2^, intravenous drip for 1 hour, d1; paclitaxel liposomes 135–175 mg/m^2^, intravenous drip for 3 hours, d1; albumin paclitaxel 260 mg/m^2^, intravenous drip for 30 minutes, d1; rh-endostatin 15 mg/m^2^, intravenous pumping for 24 hours, d1-7; bevacizumab 15 mg/kg, intravenous drip, d1. The above protocols were repeated every 21 days. Efficacy was evaluated every 2 cycles, and adverse reactions were evaluated every cycle. All patients received treatment for at least 2 cycles.

### 2.5. Dose adjustment and prevention of adverse reactions

The dosage of drugs could be adjusted in appropriate proportions according to the occurrence of adverse reactions. All carboplatin-based combination regimens were given prophylactic antiemetic therapy before application. Dexamethasone was given before docetaxel and lasted for 3 days. Dexamethasone, diphenhydramine and cimetidine were given before paclitaxel liposomes to prevent allergic reactions.

### 2.6. Study endpoints

The primary endpoints of this study were objective response rate (ORR) and progression-free survival (PFS). PFS defined as the time from initiation of carboplatin-based treatment to the occurrence of disease progression, tumor-related death, or death from any other cause. Secondary endpoints included DCR and adverse events. The response evaluation was carried out according to the Response Evaluation Criteria In Solid Tumors-RECIST 1.1,^[5]^ including complete response (CR), partial response (PR), stable disease (SD) and progressive disease, ORR = CR + PR, DCR = CR + PR + SD. Adverse effects were assessed according to the National Cancer Institute-Common Terminology Criteria for Adverse Events and were classified as 1 to 5 according to the severity.

### 2.7. Follow-up

The follow-up rate was 86.41% as of July 26, 2021 and the median follow-up time was 24.67 mo (95% CI 21.29–28.05 mo). Loss-in cases were processed as censored data in survival analyses.

### 2.8. Statistical analysis

SPSS 26.0 and R 4.2.1 were used for data processing. The best cutoff values of systemic immune-inflammation index (SII, platelet × neutrophil/lymphocyte), neutrophil-to-lymphocyte ratio (NLR) and platelet-to-lymphocyte ratio (PLR) were obtained based on the ROC curve. Counting data were expressed as composition ratio and comparison between groups were performed by χ^2^ test or Fisher’s exact probability method. Binary logistic regression was used for multivariate analysis of ORR and DCR. Survival analysis used the Kaplan–Meier method to draw the survival curve, and the Log-rank test was performed. The Cox proportional hazards regression model was used to perform univariate and multivariate analysis for the possible prognostic factors. *P* < .05 was considered statistically significant.

## 3. Results

### 3.1. Patient characteristics

**Figure 1.**
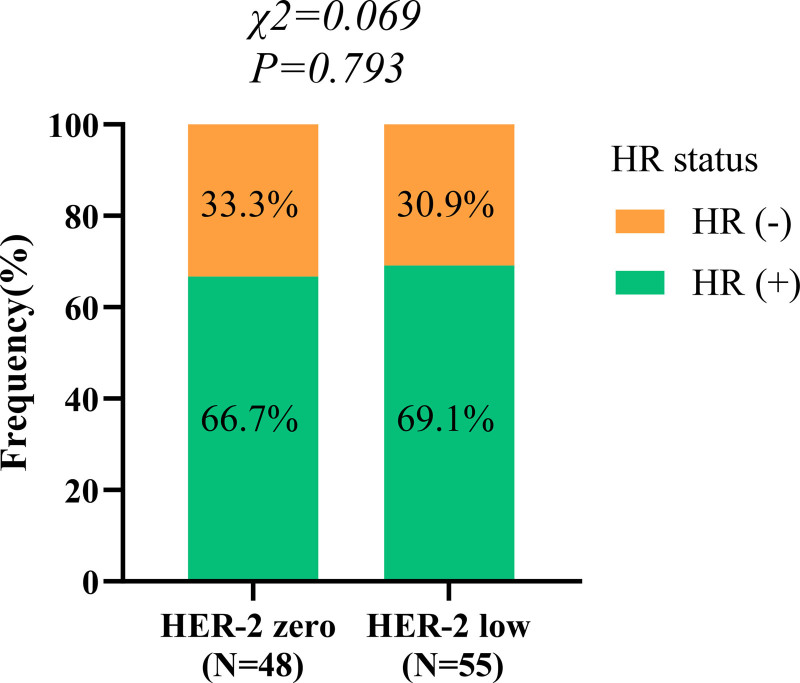
The composition ratio of different hormone receptor expression status in HER2-zero and HER2-low subgroups. HER2 = human epidermal growth factor receptor 2.

A total of 103 patients met the inclusion criteria, all of whom were female, with a median age of 50 years (range 23–75 years). There were 48 (46.60%) cases of HER2-zero breast cancers and 55 (53.40%) cases of HER2-low breast cancers (Table 1). By composition ratio analysis, different HR status and all other clinicopathological factors were well-balanced between HER2-zero and HER2-low women (Fig. 1). The best cutoff values based on ROC curves were as follows, SII > or ≤ 808.74, NLR > or ≤ 3.40, PLR > or ≤ 246.48.

**Table 1:** General characteristics of the study population

General characteristics	n (%)	General characteristics	n (%)
Median age (years)	50 (range 23–75)	Disease-free survival (DFS) (n = 64)	
<40	17 (16.50)	≤12 mo	19 (29.69)
40–60	69 (67.00)	>12 mo	45 (70.31)
>60	17 (16.50)		
ECOG		Use of anthracyclines and/or taxanes in (neo)adjuvant stage (n = 64)	
0	9 (8.74)	Have used	54 (84.38)
1	82 (79.61)	Never used	10 (15.62)
2	12 (11.65)		
Menstrual status		Sensitivity of anthracyclines and/or taxanes (n = 54)	
Premenopausal	46 (44.66)	Sensitive (DFS > 12 mo)	39 (72.22)
Postmenopausal	57 (55.34)	Insensitive (DFS ≤ 12 mo)	15 (27.78)
HR status		SII	
Positive	70 (67.96)	≤808.74	44 (42.72)
Negative	33 (32.04)	>808.74	59 (57.28)
HER2 status		NLR	
HER2-zero	48 (46.60)	≤3.40	60 (58.25)
HER2-low	55 (53.40)	>3.40	43 (41.75)
Use of anti-angiogenic agents		PLR	
Have used	20 (19.42)	≤246.48	75 (72.82)
Never used	83 (80.58)	>246.48	28 (27.18)
Initial diagnosis			
Metastatic breast cancer	39 (37.86)		
Non-metastatic breast cancer	64 (62.14)		

HER2 = human epidermal growth factor receptor 2, HR = risk ratio, NLR = neutrophil-to-lymphocyte ratio, PLR = platelet-to-lymphocyte ratio, SII = systemic immune-inflammation index.

### 3.2. Short-term efficacy

As of July 26, 2021, all 103 patients had completed at least 2 cycles of chemotherapy, and the median number of cycles of chemotherapy received by each patient was 5 cycles (range 2–24 cycles), all of which could be evaluated. Among them, 1 cases (0.97%) were obtained CR, 43 cases (41.75%) were PR, 33 cases (32.04%) were SD and 26 cases (25.24%) were progressive disease. The ORR of the whole group was 42.72% and the DCR was 74.76%.

### 3.3. Comparison of short-term efficacy among subgroups with different clinicopathological features

The ORR of HER2-low patients was significantly higher than HER2-zero patients (56.4% vs 27.1%, *P* = .003). When stratified by HR status, different results were observed in HR-positive and HR-negative subgroups (HR(+): HER2-low 50.0% vs HER2-zero 34.4%, *P* = .188; HR(-): HER2-low 70.6% vs HER2-zero 12.5%, *P* = .001). Grouping according to age, ECOG score, menstrual status, HR status, DFS for initial diagnosis of non-metastatic breast cancer, use of anthracyclines and/or taxanes in the (neo)adjuvant stage, sensitivity of anthracycline and/or taxanes, use of antiangiogenic drugs, SII, NLR and PLR, there was no statistically significant difference in ORR (all *P* > .05) in each group. DCR varied significantly in different age groups (*P* = .042), whereas there were no significant differences in DCR (all *P* > .05) in remaining groups (Table 2).

**Table 2:** Analysis of differences of ORR and DCR among subgroups with different clinicopathological features

General characteristics	N	ORR (n, %)	χ^2^	*P*	DCR (n, %)	χ^2^	*P*
Age (years)			2.648	.266		5.780	.042
<40	17	8 (47.1)			10 (58.8)		
40–60	69	26 (37.7)			51 (73.9)		
>60	17	10 (58.8)			16 (94.1)		
ECOG			0.664	.717		0.849	.649
0	9	5 (55.6)			8 (88.9)		
1	82	34 (41.5)			60 (73.2)		
2	12	5 (41.7)			9 (75.0)		
Menstrual status			0.020	.889		1.187	.276
Premenopausal	46	20 (43.5)			32 (69.6)		
Postmenopausal	57	24 (42.1)			45 (78.9)		
HR status			0.002	.967		1.684	.194
Positive	70	30 (42.9)			55 (78.6)		
Negative	33	14 (42.4)			22 (66.7)		
HER2 status			8.980	.003		3.118	.077
HER2-zero	48	13 (27.1)			32 (66.7)		
HER2-low	55	31 (56.4)			45 (81.8)		
HR(+)/HER2(-) (n = 70)			1.732	.188		1.570	.210
HER2-zero	32	11 (34.4)			23 (71.9)		
HER2-low	38	19 (50.0)			32 (84.2)		
HR(-)/HER2(-) (n = 33)			11.386	.001		1.517	.218
HER2-zero	16	2 (12.5)			9 (56.3)		
HER2-low	17	12 (70.6)			13 (76.5)		
Initially diagnosed non-metastatic breast cancer (n = 64)			2.197	.138		0.001	.977
DFS ≤ 12 mo	19	11 (57.9)			14 (73.7)		
DFS > 12 mo	45	17 (37.8)			33 (73.3)		
Use of anthracyclines and/or taxanes in (neo)adjuvant stage (n = 64)			-	.737		-	1.000
Have used	54	23 (42.6)			40 (74.1)		
Never used	10	5 (50.0)			7 (70.0)		
Sensitivity of anthracyclines and/or taxanes (n = 54)			2.574	.109		-	.733
Sensitive (DFS > 12 mo)	39	14 (35.9)			28 (71.8)		
Insensitive (DFS ≤ 12 mo)	15	9 (60.0)			12 (80.0)		
Use of anti-angiogenic agents			0.053	.818		1.252	.263
Have used	20	9 (45.0)			13 (65.0)		
Never used	83	35 (42.2)			64 (77.1)		
SII			0.788	.375		0.933	.334
≤808.74	44	21 (47.7)			35 (79.5)		
>808.74	59	23 (39.0)			42 (71.2)		
NLR			0.306	.580		3.636	.057
≤3.40	60	27 (45.0)			49 (81.7)		
>3.40	43	17 (39.5)			28 (65.1)		
PLR			0.771	.380		0.226	.635
≤246.48	75	34 (45.3)			57 (76.0)		
>246.48	28	10 (35.7)			20 (71.4)		

HER2 = human epidermal growth factor receptor 2, HR = risk ratio, NLR = neutrophil-to-lymphocyte ratio, ORR = objective response rate, PLR = platelet-to-lymphocyte ratio, SII = systemic immune-inflammation index.

### 3.4. Analysis of relevant factors affecting the short-term efficacy

First of all, univariate analysis was performed to select clinicopathological features probably influencing short-term efficacy. The results showed that HER2-zero was an independent risk factor of ORR (OR 3.478, 95% CI 1.516–7.977, *P* = .003), especially in the HR-negative subgroup (Table 3). The remaining clinical features were not associated with ORR (all *P* > .05). In this study, no relevant factors related to DCR were found (all *P* > .05).

**Table 3:** Univariate analysis of ORR

Influencing factors	OR (95% CI)	*P*
HER2 status		.003
HER2-zero	1.000	
HER2-low	3.478 (1.516–7.977)	
HR(+)/HER2(-) (n = 70)		.190
HER2-zero	1.000	
HER2-low	1.909 (0.725–5.025)	
HR(-)/HER2(-) (n = 33)		.002
HER2-zero	1.000	
HER2-low	16.800 (2.744–102.866)	

HER2 = human epidermal growth factor receptor 2, HR = risk ratio, ORR = objective response rate.

### 3.5. Survival analysis

**Figure 2.**
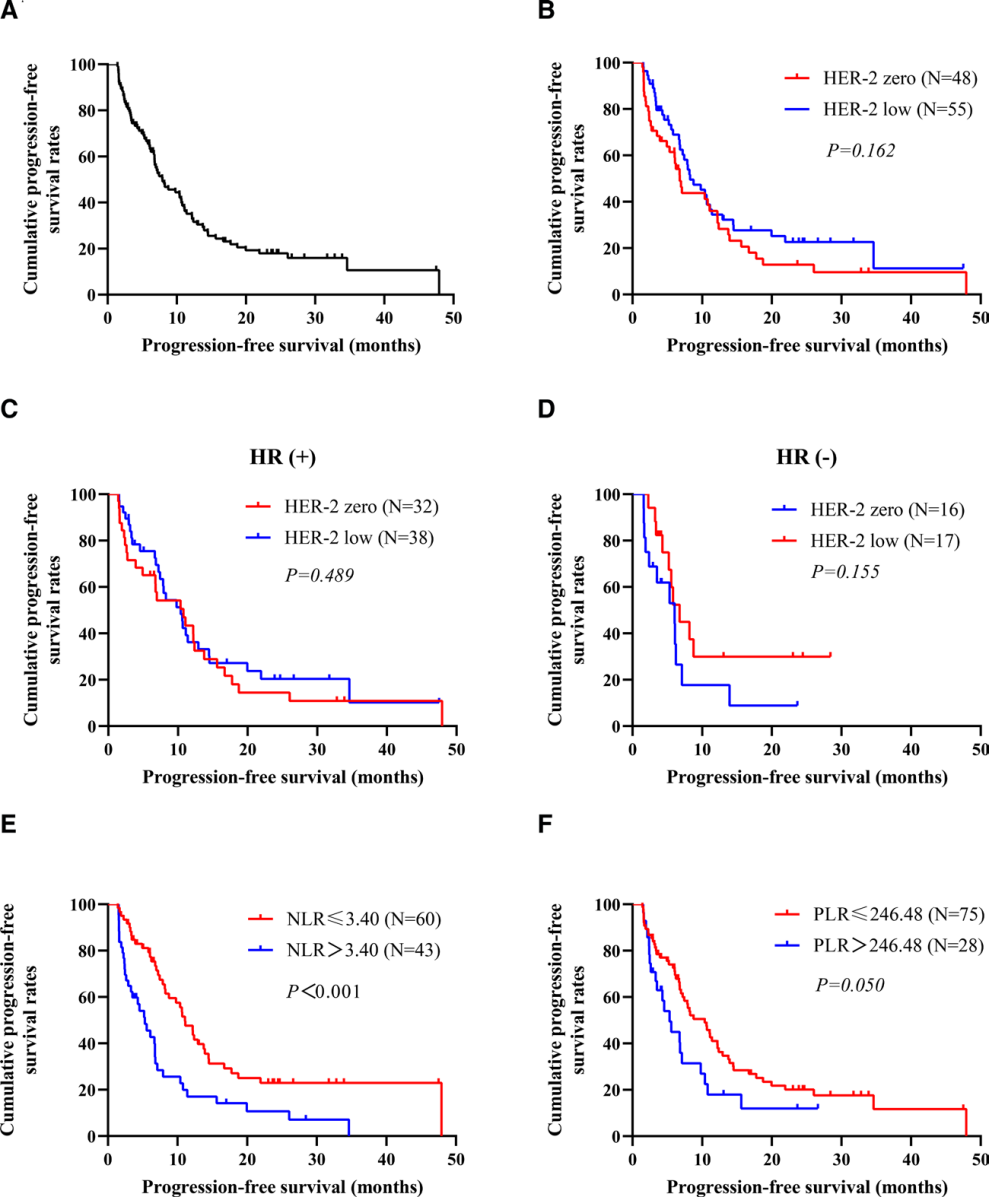
Kaplan–Meier plot of PFS for (A) The whole group, (B) Different HER2 status, (C) Different HER2 status in HR(+) subgroup, (D) Different HER2 status in HR(−) subgroup, (E) Different NLR values, and (F) Different PLR values. HER2 = human epidermal growth factor receptor 2, HR = risk ratio, NLR = neutrophil-to-lymphocyte ratio, PFS = progression-free survival, PLR = platelet-to-lymphocyte ratio.

The median follow-up was 24.67 mo (95% CI 21.29–28.05 mo), and the follow-up rate was 86.41%. By the end of follow-up, a total of 77 (74.76%) patients had disease progression. The mPFS of the whole group was 7.93 mo (95% CI 5.17–10.70 mo) (Fig. 2A).

### 3.6. Subgroup analysis of survival

Survival analysis showed that the survival advantage was no longer significant in HER2-low patients compared to HER2-zero patients (*P* = .162), regardless of HR status (Fig. 2B–D). In addition, the mPFS was significantly longer in patients with low NLR scores than those with high NLR scores (*P* < .001) (Fig. 2E). Similarly, there seemed to be a better trend of progression-free survival in patients with low PLR scores than those with high PLR scores (*P* = .050) (Fig. 2F). However, there were no statistically significant differences in mPFS between subgroups based on age, ECOG score, menstrual status, HR status, DFS for initial diagnosis of non-metastatic breast cancer, use of anthracyclines and/or taxanes in the (neo)adjuvant stage, sensitivity of anthracycline and/or taxanes, use of antiangiogenic drugs, or different SII scores (all *P* > .05).

### 3.7. Univariate and multivariate analysis of survival

**Figure 3.**
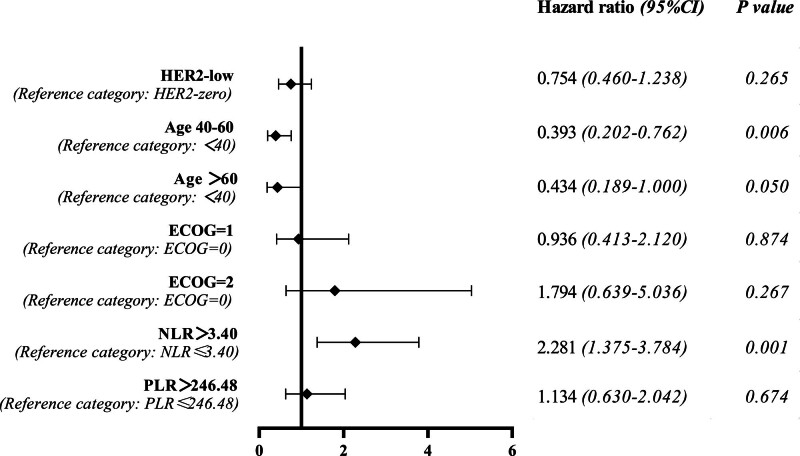
Forest plot based on multivariate analysis of PFS. PFS = progression-free survival.

The results of univariate analysis showed that age (*P* = .036) and NLR (*P* = .001) might be prognostic factors affecting mPFS. However, HER2-low status was not observed to be associated with prolonged survival (*P* = .164), regardless of HR status (Table 4). The result of multivariate analysis showed that young breast cancer (age < 40) (*P* = .006) and high NLR values (*P* = .001) were prognostic risk factors affecting mPFS, after adjusting for confounding factors such as ECOG, HER2 status and PLR (Fig. 3).

**Table 4:** Univariate analysis of PFS

Influencing factors	HR (95% CI)	*P*
Age (years)		.094
<40	1.000	
40–60	0.510 (0.272–0.958)	.036
>60	0.486 (0.219–1.079)	.076
ECOG		.090
0	1.000	
1	1.273 (0.580–2.795)	.548
2	2.486 (0.959–6.445)	.061
Menstrual status		.646
Premenopausal	1.000	
Postmenopausal	0.900 (0.573–1.413)	
HR status		.326
Negative	1.000	
Positive	0.777 (0.470–1.286)	
HER2 status		.164
HER2-zero	1.000	
HER2-low	0.726 (0.463–1.139)	
HR(+)/HER2(-) (n = 70)		.489
HER2-zero	1.000	
HER2-low	0.828 (0.485–1.414)	
HR(-)/HER2(-) (n = 33)		.161
HER2-zero	1.000	
HER2-low	0.546 (0.234–1.273)	
Initially diagnosed non-metastatic breast cancer (n = 64)		.945
DFS > 12 mo	1.000	
DFS ≤ 12 mo	1.022 (0.549–1.902)	
Use of anthracyclines and/or taxanes in (neo)adjuvant stage (n = 64)		.128
Never used	1.000	
Have used	1.955 (0.825–4.635)	
Sensitivity of anthracyclines and/or taxanes (n = 54)		.869
Sensitive (DFS > 12 mo)	1.000	
Insensitive (DFS ≤ 12 mo)	1.060 (0.533–2.107)	
Use of anti-angiogenic agents		.688
Never used	1.000	
Have used	0.881 (0.474–1.636)	
SII		.228
≤808.74	1.000	
>808.74	1.327 (0.838–2.102)	
NLR		.001
≤3.40	1.000	
>3.40	2.219 (1.407–3.501)	
PLR		.053
≤246.48	1.000	
>246.48	1.656 (0.994–2.758)	

HER2 = human epidermal growth factor receptor 2, HR = risk ratio, NLR = neutrophil-to-lymphocyte ratio, PFS = progression-free survival, PLR = platelet-to-lymphocyte ratio, SII = systemic immune-inflammation index.

### 3.8. Safety

This study aimed to evaluate the toxicity of laboratory indicators such as hematological toxicity, liver and kidney toxicity. The incidence of grade 3 to 4 adverse reactions was higher in neutropenia (15.53%), anemia (15.53%), and leukopenia (11.65%), but lower in thrombocytopenia (5.83%) and elevated aminotransferases (2.91%), moreover, no grade 3 to 4 elevated creatinine was reported (Table 5). Adverse reactions could return to normal after treatment, and no treatment-related deaths occurred in the whole group.

**Table 5:** Occurrence of adverse reactions in the whole process

Adverse events	Grades					
	1	2	3	4	1–4	3–4
Leukopenia	32 (31.07)	33 (32.04)	9 (8.74)	3 (2.91)	77 (74.76)	12 (11.65)
Neutropenia	26 (25.24)	28 (27.18)	12 (11.65)	4 (3.88)	70 (67.96)	16 (15.53)
Anemia	26 (25.24)	38 (36.89)	16 (15.53)	0 (0.00)	80 (77.67)	16 (15.53)
Thrombocytopenia	3 (2.91)	8 (7.77)	4 (3.88)	2 (1.94)	17 (16.50)	6 (5.83)
Elevated transaminase	46 (44.66)	5 (4.85)	3 (2.91)	0 (0.00)	54 (52.43)	3 (2.91)
Elevated creatinine	1 (0.97)	0 (0.00)	0 (0.00)	0 (0.00)	1 (0.97)	0 (0.00)

## 4. Discussion

Platinum drugs act on the DNA double strands of tumor cells, cross-linking with DNA to break the double strands, thereby interfering with DNA synthesis, mediating apoptosis of tumor cells and exerting anti-tumor effects. Meta-analysis^[6]^ supported the overall efficacy of platinum-based chemotherapy was superior to non-platinum-based chemotherapy for the first-line treatment of metastatic breast cancer (OR 1.47, 95% CI 1.23–1.76, *P* = .0001). In this study, 103 patients who received first-line carboplatin-based regimens obtained an overall ORR of 42.72% and an mPFS of 7.93 m, which was consistent with previous clinical studies,^[7–11]^ suggesting that the carboplatin-based regimens might have good efficacy.

For HER2-negative breast cancer, the advantages of platinum drugs in TNBC have been confirmed by many clinical studies.^[12–17]^ Current guidelines^[2,3]^ generally recommend platinum drugs for patients with TNBC carrying BRCA1/2 mutations, as the homologous recombination repair pathway after DNA damage is blocked due to BRCA1/2 mutations,^[18,19]^ which can improve the sensitivity of platinum drugs to tumor cells. For HR-positive, HER2-negative patients with visceral crisis that require rapid response in the short term, or resistance to endocrine therapy, anthracycline and/or taxanes-based chemotherapy are preferred, and this study found that carboplatin-based chemotherapy also have good efficacy.^[20–22]^

Previously, based on IHC and ISH results, HER2 status was only classified into positive and negative. HER2-positive was once considered a poor prognostic factor, but with the emergence and rapid development of anti-HER2 drugs, such as trastuzumab, the prognosis for survival of HER2-positive patients has been significantly improved.^[23,24]^ In recent years, as HER2-low status has continued to exhibit its clinical and biological features distinguishing from HER2-zero, studies based on HER2-negative breast cancer have gradually been further divided into HER2-low and HER2-zero, and a growing number of researchers have begun to explore whether HER2-low can be regarded as an independent molecular subtype, thereby guiding the clinical therapy. A considerable number of studies have pointed out^[25]^ that HRs are detected in HER2-low tumors at a higher rate than HER2-zero. Schettini et al^[26]^ found that compared with HER2-zero tumors, Luminal-related genes were significantly upregulated in HER2-low tumors, while Basal-like genes and proliferation-related genes were significantly downregulated in HER2-low tumors. It is speculated that there is an association between HR status and HER2 expression status, and endocrine therapy may play an important role in HER2-low tumors. For general HR-positive, HER2-negative breast cancer, endocrine therapy with CDK4/6 inhibitor is a standard therapy. Nevertheless, if we further explore whether HER2-low and HER2-zero statuses affect the efficacy and prognosis of CDK4/6 inhibitor combined with endocrine therapy, the conclusions of current studies are still controversial.^[27–29]^ In this regard, there has been a multicenter retrospective study revealing that HER2-low status did not have a significant effect on response rate and PFS, but in the patients with recurrent metastatic disease, the PFS of HER2-low showed a tendency to be prolonged,^[27]^ and this result also predicts that further prospective studies are imminent to provide more definitive evidence. However, there are still cases of resistance to endocrine therapy or the urgent need for short-term clinical response so that CDK4/6 inhibitors and endocrine therapies are inappropriate for use. In any case, for HER2-negative tumors (regardless of HR status) receiving chemotherapy alone, such studies are lacking in whether the effects of chemotherapy differ between HER2-low and HER2-zero tumors. Most studies have shown that the survival advantage of HER2-low tumors over HER2-zero tumors receiving chemotherapy alone was only manifested in HR-positive breast cancer,^[25,30,31]^ while not observed in TNBC. This study yielded mixed results. Based on the premise that clinicopathological features such as age and HR status were balanced in HER2-zero and HER2-low patients, HER2-low patients (regardless of HR status) who received first-line carboplatin-based chemotherapy were more likely to benefit than HER2-zero, and this trend was more significant in TNBC. Further exploring the relationship between chemotherapy and HER2-low status, and summarizing the survival data of related studies in Table 6, the results show that compared with HER2-zero patients, whether 1-2 lines of chemotherapy can benefit HER2-low patients in PFS and OS has not been consistent,^[30,32–35]^ but some studies have proposed that HER2-low patients receiving first-line chemotherapy could significantly extend the survival compared with HER2-zero patients.^[30,33]^ In recent years, with the deepening of research on ADCs, DB04 have reported that trastuzumab deruxtecan conferred survival benefits in both the total HER2-low population and the HR-positive/negative subgroups compared with treatment of physician’s choice, thus opening up new treatment ideas for HER2-low breast cancer.^[4]^ However, considering the accessibility of ADCs and the economic factors of patients, there is still a need for more convenient, economical and effective treatment options to provide patients with choices on the front line. Based on the results of this study, it is suggested that carboplatin-based chemotherapy may benefit HER2-low patients, especially TNBC. For TNBC patients of HER2-zero, more possible therapeutic targets such as immune checkpoints should be sought through genetic testing. The differential effects of chemotherapy on HER2-low tumors may partly explain why HER2-low tumors are sensitive to ADCs rather than traditional anti-HER2 therapy.^[36]^ The mechanisms need to be further explored and studied. More strategies and methods based on novel anti-HER2 therapy urgently need to be discovered and applied to HER2-low advanced breast cancer.

**Table 6:** Summary of survival studies of HER2-low versus HER2-zero advanced breast cancer with chemotherapy

	lines	PFS (months)				OS (months)			
		HER2-zero	HER2-low		*P*	HER2-zero	HER2-low		*P*
			1+	2+/ISH-			1+	2+/ISH-	
Alexander Hein 2021^[32]^	1-2	4	3.5	2	.63	13	11	7	.055
Ombline de Calbiac 2022^[33]^	1	4.6	5.3		.009	13.3	15.6		.04
Paolo Tarantino 2022^[34]^	1	–	–		.76	–	–		.49
Yiqun Li 2021^[35]^	NA	–	–		–	29.9	29.5		.718
						29.9	27.2	42.9	.139
Changchuan Jiang 2022^[30]^	1	–	–		–	28.4	36.7		<.001

HER2 = human epidermal growth factor receptor 2, PFS = progression-free survival.

The body’s inflammatory response and immune status have been shown to be closely related to the processes of tumor proliferation, metastasis, and angiogenesis.^[37]^ SII, NLR, PLR, and other parameters based on the counts of platelets, neutrophils, lymphocytes and other inflammatory cells can well reflect the immune inflammatory state of the body, and have been confirmed to have prognostic effects in many cancers including breast cancer.^[38,39]^ Studies have reported that SII could be used as an independent predictor of DFS and OS in breast cancer, and higher SII predicted worse survival, but these studies were mostly limited to stage I-III breast cancer that received neoadjuvant/adjuvant therapies.^[39–41]^ In a limited number of studies on metastatic breast cancer, SII was found inadequate as a prognostic predictor for OS.^[42]^ Nevertheless, this study found that in patients with metastatic breast cancer, lower NLR before initiation of treatment predicted extended PFS. Meanwhile, SII and PLR also had certain predictive value for prognosis. Considering that clinical hematology testing is rapid, efficient, cost-effective and practical, it may be valuable to use inflammation index to predict the efficacy and prognosis of patients with metastatic breast cancer before treatment. Considering that inflammatory index may be affected by metabolic syndrome, hypercholesterolemia, abnormal thyroid function, smoking and alcohol consumption,^[41]^ it is necessary to further expand the sample size and eliminate the influence of confounding factors before conducting research.

In terms of safety, the incidences of grade 3 to 4 leukopenia, neutropenia, anemia, thrombocytopenia, elevated aminotransferases and elevated creatinine in this study were 11.65%, 15.53%, 15.53%, 5.83%, 2.91%, and 0.00%, respectively, which were basically consistent with the previous reports,^[7–11,43]^ suggesting that the adverse reactions of carboplatin-based regimens were controllable and well tolerated.

However, the results showed that carboplatin-based regimens had good efficacy and safety in HER2-low advanced breast cancer. Secondly, as of the end of follow-up, the mortality of the whole group did not reach 50%, and the overall survival could not be evaluated. Finally, most HR-positive/HER2-low patients in this study did not receive endocrine therapy before first-line rescue chemotherapy, so the efficacy and safety of using carboplatin-based chemotherapy after progression following endocrine therapy need to be further verified.

## 5. Conclusion

Based on the results of this study, the first-line carboplatin-based chemotherapy is quite active and tolerable in patients with HER2-low advanced breast cancer, that higher response rates can be achieved. In cases where CDK4/6 inhibitors are inappropriate for use due to resistance to endocrine therapy or the urgent need for short-term clinical response, chemotherapy remains important. Moreover, when it is necessary to consider the accessibility of ADCs and the economics of patients, carboplatin-based chemotherapy may be provided to HER2-low patients as a more convenient, cost-effective and efficient option on the front line. For TNBC patients of HER2-zero, more possible therapeutic targets such as immune checkpoints should be sought through genetic testing. In addition, forecasting the efficacy and prognosis via inflammatory index such as NLR before the commencement of the treatment could enhance the precision and efficiency of carboplatin-based regimens.

## Author contributions

**Conceptualization:** Jingxin Li, Weimin Xie.

**Data curation:** Jingxin Li, Yijing Tang, Qianying Chen, Sen Lei.

**Formal analysis:** Jingxin Li, Yijing Tang, Qianying Chen.

**Funding acquisition:** Jingxin Li.

**Investigation:** Jingxin Li.

**Methodology:** Jingxin Li, Weimin Xie.

**Project administration:** Yongkui Lu.

**Software:** Jingxin Li.

**Supervision:** Yongkui Lu, Aihua Tan, Weimin Xie.

**Writing – original draft:** Jingxin Li.

**Writing – review & editing:** Weimin Xie.
